# A late-onset Ornitin Transcabamylase deficiency case as an organic psychosis

**DOI:** 10.1192/j.eurpsy.2022.2048

**Published:** 2022-09-01

**Authors:** M.J. Mateos Sexmero, J.A. Blanco

**Affiliations:** 1 Hospital Clínico Universitario, Psiquiatría, Valladolid, Spain; 2 University Clinical Hospital of Valladolid, Psychiatry, Valladolid, Spain

**Keywords:** OTC deficiency, Organic psychosis, Psychosis

## Abstract

**Introduction:**

Ornithine transcarbamylase (OTC) deficiency is the most frequent congenital defect among the urea cycle enzymatic disorders, due to mutations a!ecting the OTC gen (Xp21.1) that are inherited with an X-linked pattern. As it happens with sex-linked genetic disorders, late-onset OTC deficiency is more prevalent among women, so that females may be asymptomatic over the years and manifest symptoms only when they are submitted under severe metabolic stress, such as pregnancy, infections or new medications. The enzymatic defect involves a blockage a!ecting the main biochemical route that converts ammonia into urea. This leads to analytic hyperammonemia and the outburst of gastrointestinal, neurological and psychiatric symptoms with variable severity.

**Objectives:**

Expounding the importance of inborn errors of metabolism as possible causes of a psychotic episode.

**Methods:**

Describing the case, supporting our data with a bibliographic research made on PubMed.

**Results:**

We describe a psychiatric adult-onset OTC deficiency in a 37-year-old woman with borderline intellectual functioning and a psychotic episode in the context of an infection that was wrongly diagnosed at first as schizophrenia, until the genetic study was carried out. The woman’s familiar history shown an OTC deficiency among some family members, a mutation- carrier sister and at least two male children death by the first month of life.

**Conclusions:**

Organic psychosis can be caused by a large number of medical diseases. A di!erential diagnosis of possible cerebral, toxic or metabolic causes of psychosis is necessary to avoid mistakes in diagnosis.

**Disclosure:**

No significant relationships.

